# Reply to: Critical COVID-19 and neurological dysfunction - a direct comparative analysis between SARS-CoV-2 and other infectious pathogens

**DOI:** 10.5935/2965-2774.20230104resp-en

**Published:** 2023

**Authors:** Ana Teixeira-Vaz, José Artur Paiva

**Affiliations:** 1 Physical Medicine and Rehabilitation Department, Centro Hospitalar Universitário de São João, Faculdade de Medicina, Universidade do Porto - Porto, Portugal; 2 Intensive Care Medicine Department, Centro Hospitalar Universitário de São João, Faculdade de Medicina, Universidade do Porto - Porto, Portugal

## TO THE EDITOR

We thank Dr. Finsterer and Dr. Scorza for their interest in and praise for our study entitled “Critical COVID-19 and neurological dysfunction - a direct comparative analysis between SARS-CoV-2 and other infectious pathogens”.^(1)^

It is pointed out that our study’s clinical neurological examination was incomplete. It is true that, for the strict sake of the study, the researchers did not perform a detailed neurological examination of the included patients, but instead focused on the assessment of signs of corticospinal tract dysfunction (CSTD), in accordance with the rationale of the study. Our research primary goal was not to fully characterize the neurological status of acute respiratory distress syndrome (ARDS) patients but to identify whether SARS-CoV-2 is more frequently associated with signs of CSTD (and other neurological signs, symptoms, and syndromes) than other pathogens causing severe respiratory failure. As reported by Parsons et al.^(2)^ and referenced in our paper, corticospinal tract lesions are the most common lesions of the white matter depicted in COVID-19 patients; these lesions were the focus of our study. Accordingly, our sample size was calculated based on the use of deep tendon reflex responses as the main outcome variable. Nonetheless, all patients were submitted to daily full neurological examinations during their stay in the intensive care unit (ICU); these examinations were performed by the attending intensive care physicians in accordance with the standard of clinical practice of the Intensive Care Medicine Department. All relevant data (clinical, laboratory, and complementary diagnostic tests) regarding the presence of neurological signs, symptoms, and syndromes during the ICU stay were included in the electronic clinical records and retrieved by the researchers.

False-negative reverse transcription polymerase chain reaction (RT-PCR) results among COVID-19 patients may be caused by observer errors, but they are mainly caused by low viral RNA levels in the later stages of disease after the infection has cleared. In fact, clinicians should be aware that patients with COVID-19 can have negative RT-PCR SARS-CoV-2 test results in the later stages of infection.^(3)^ The viral load of SARS-CoV-2 has been reported to change with time, with a high viral load observed in the first week after onset of illness and a low viral load observed two weeks after onset. The main conclusion of the paper used as a reference by the authors of the letter is that consecutive negative RT-PCR results from respiratory specimens may not be a suitable criterion for viral clearance, not for diagnosis.^(4)^ In fact, serial RT-PCR testing can effectively rule out the diagnosis of COVID-19; after multiple negative RT-PCR tests, other diagnoses should be considered. In our population, all cases of ARDS were assessed through nasal/pharyngeal swabs. In cases of negativity and in the absence of other causes, the test was repeated, and, whenever possible, a bronchoalveolar lavage sample was obtained for diagnostic microbiological tests that also included RT-PCR SARS-CoV-2.

The letter stated that the study should detail how many of the included patients were suspected of having not only SARS-CoV-2 infections but also other types of infections. These data are available in table 2 of our paper,^(1)^ which shows that 85% of COVID-19 cases had overlapping infections (superinfections) while only 26% of the patients in the control group had overlapping infections. Although this finding is not formally discussed, it is in line with the results reported by Nokhodian et al.^(5)^

Regarding the definition of seizures, we stated that a seizure was considered a change in the level of consciousness, behavior, memory, or feelings related to uncontrolled and/or abnormal electrical activity of the brain. It is well recognized that patients with seizures may present not only alterations in the level and content of consciousness but also other clinical signs, such as tingling, jerking movements, muscle tightening, rapid eye movement, aura sensation, hallucinations, alterations in smells, taste, tactile sensation or sight.^(6)^ Therefore, we did not consider that seizures were only present when there was a change in consciousness, and a more in-depth definition was adopted. Whenever a suspicion of seizure was raised, an electroencephalogram was performed, and clinical and electrophysiological assessments were performed by a neurophysiologist in collaboration with the intensivist. In our sample, only two patients developed seizures during their ICU stay; those patients underwent an electroencephalogram and received tailored therapy.

Concerning the definition of peripheral neuropathies, in our paper, we stated that these neuropathies included disorders of peripheral nerve cells and fibers, including mononeuropathies, multifocal neuropathies and polyneuropathies (which include small and large fiber neuropathies). Nonetheless, we agree that polyradiculitis and plexopathy could have been included for a broader definition. Moreover, the authors of the letter suggest that it should be known how many of the enrolled patients had neuropathy that was due to critical illness and not to SARS-CoV-2 infection. We highlight that in real-life critical care practice, the differential diagnosis between these entities is extremely difficult, requiring invasive complementary diagnostic studies. Additionally, the differential diagnosis between these entities has not been proven to be helpful for the definition of treatment and prognosis. Furthermore, only two patients in our population had peripheral neuropathies. Further studies aiming to analyze this issue would be very interesting.

Moreover, Dr. Finsterer and Dr. Scorza state that it would be important to know how many of the included patients had a stroke due to venous sinus thrombosis (VST). Indeed, we acknowledge that the risk of VST is higher among COVID-19 patients.^(7)^ As described in table 2 of our article,^(1)^ none of the included patients had ischemic or hemorrhagic strokes; we only observed one case of transient ischemic attack (in which imagiological data were not compatible with VST).

In line with the letter, we agree that stroke, encephalitis, epilepsy, myelitis, and neuropathy cannot be diagnosed solely using a clinical exam, and we do not state, in any part of our paper, that instrumental examinations would be unnecessary to perform these diagnoses. Indeed, we retrieved the information on these neurological syndromes from electronic clinical records, where the means to conclude that diagnosis included subsidiary examinations. Nonetheless, that was not the paper’s main focus; therefore, in our Results section, we do not detail the instrumental evaluations that each patient performed.

We acknowledge the limitations of our paper, and we thank the authors of the letter for raising questions that helped us to clarify certain aspects of our study as well as aspects of the routine practice of our Intensive Care Medicine Department activity and clinical records, which includes permanent intensivist-based multidisciplinary practice with at least two full patients’ clinical assessments and two multidisciplinary ICU rounds per day.
